# Supplementary material for: Unraveling the pathological biomineralization of monosodium urate crystals in gout patients
Source: Commun Biol. 2024 Jul 7;7:828. doi: 10.1038/s42003-024-06534-6 (PMC11228021; doi:10.1038/s42003-024-06534-6)
Supplement: Supplementary file 2 — Supplementary Information [file 42003_2024_6534_MOESM2_ESM.pdf]

Supplementary Information

Unraveling the pathological biomineralization of monosodium urate crystals in gout patients

Carlos Rodriguez-Navarro,[a]* Kerstin Elert,[a,b] Aurelia Ibañez-Velasco,[a] Luis Monasterio-Guillot,[a] Mariano Andres,[c,d] Francisca Sivera,[c,e] Eliseo Pascual[c,d] and Encarnación Ruiz-Agudo[a]

[a]Dept. Mineralogy and Petrology, University of Granada, Fuentenueva s/n, 18002 Granada, Spain

[b]Escuela de Estudios Arabes, Consejo Superior de Investigaciones Científicas (EEA-CSIC), C. Chapiz 22, 18010, Granada, Spain

[c]Dept. Clinical Medicine, Miguel Hernandez University, CN 332 s/n, 03550 Alicante, Spain.

[d]Dept. Rheumatology, Dr. Balmis General University Hospital, Alicante Institute for Health and Biomedical Research, Av. Pintor Baeza s/n, 03010 Alicante, Spain.

[e]Dept. Rheumatology, Elda General University Hospital, Carretera Elda-Sax s/n, 03600, Elda, Spain.

* Corresponding author: carlosrn@ugr.es

This file includes:

Supplementary Figures S1-S6

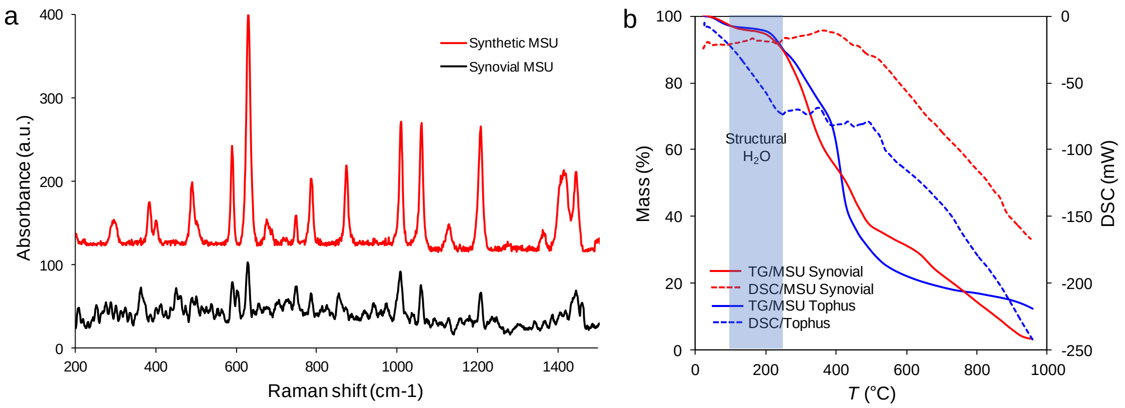

Fig. S1. **Characterization of biotic MSU from the synovial fluid of gouty patients**. a) Raman spectrum of biotic MSU. The spectrum of an abiotic control (precipitated using the titration route) is included for comparison. Note that the main bands of MSU are present in biotic MSU. However, due to its relatively low concentration in SF, the intensity of the bands is reduced; b) TG and DSC traces of biotic MSU (from SF and from a tophus). The final weight loss due to dehydration (from 25-200 °C) and urate decomposition (200-950 °C) are observed. However, they are less defined in the SF MSU, as compared with the tophus derived MSU, due to the presence of abundant organics in the former. This is why the final weight loss is higher in SF MSU. The endothermic band corresponding to dehydration and organic/urate decomposition (in air) are better defined in the case of tophus-derived MSU.

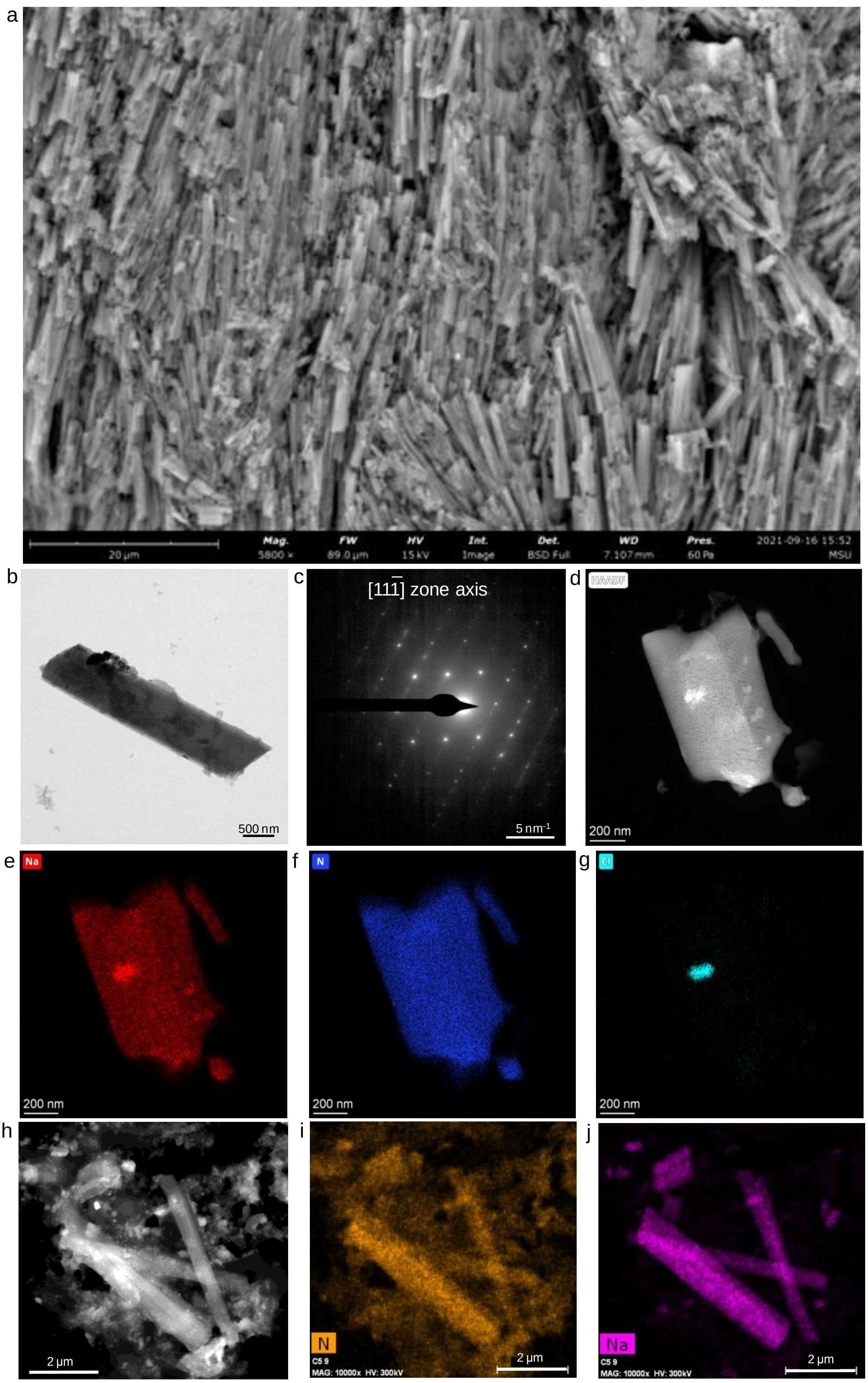

Fig. S2. **Electron microscopy analysis of human MSU**. a) SEM image of oriented aggregates of MSU crystals in a tophus; b) TEM image of a MSU crystal; c) SAED pattern of the MSU crystals in (b). d) HAADF image of MSU, and corresponding Na

(e), N (f) and Cl (g) EDS maps. Note that the bright central spot in (d) corresponds to NaCl, presumably formed during drying of the SF sample; h) Aggregate of MSU crystals as demonstrated by their N (i) and Na (j) EDS maps. Note that the shapeless material surrounding the crystals does not include Na, but it is N-rich, which suggests they are proteins from the SF.

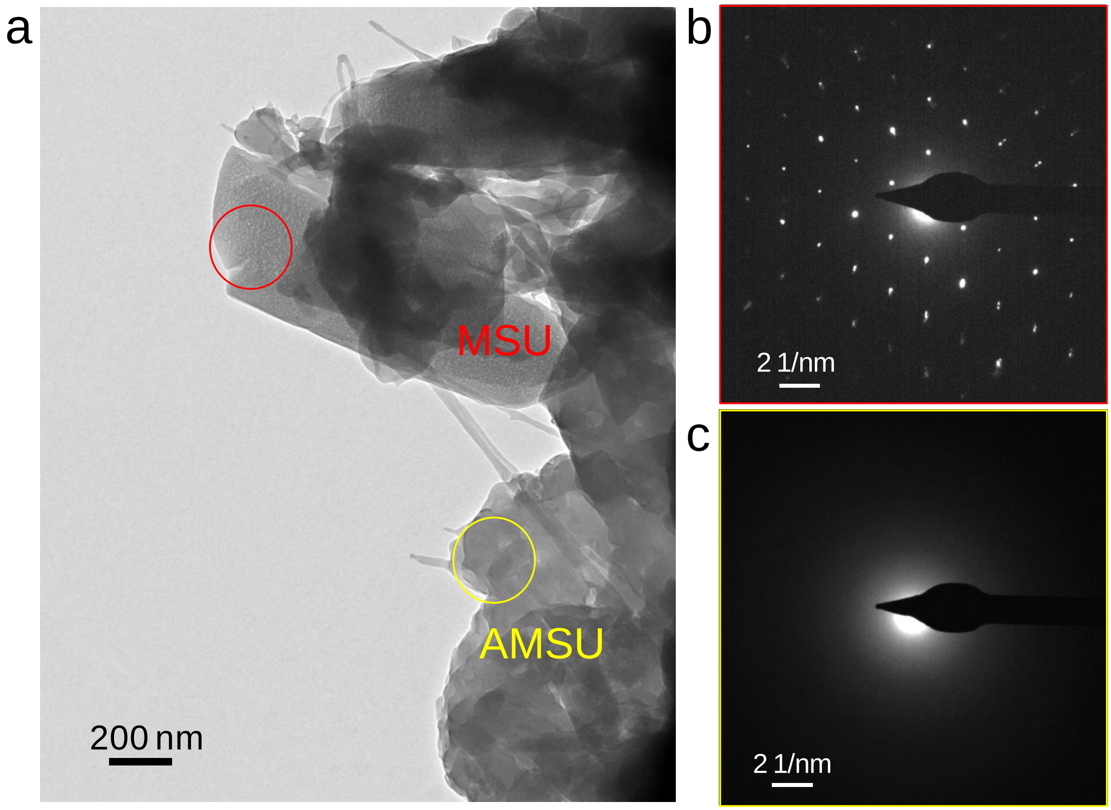

Fig. S3. AMSU and MSU in a gouty patient synovial fluid. a) TEM image of AMSU and MSU; b) SAED of MSU (red circle in (a)); and c) SAED of AMSU (yellow circle in (a)).

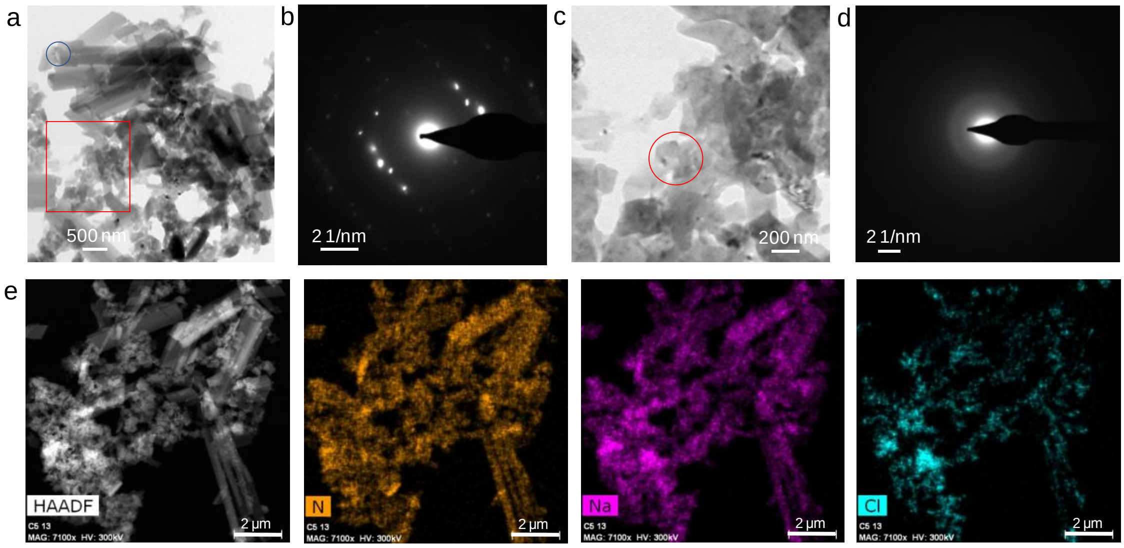

Fig. S4. **TEM-EDS analysis of AMSU and MSU in the synovial fluid of a gouty patient**. a) TEM bright field image of an aggregate of sodium urate precipitates. The prismatic/blade-like elongated crystals are MSU as shown by their SAED pattern (b) corresponding to the red circle in (a). c) Enlarged view of the red squared area in (a) showing nanogranular aggregates of AMSU, as demonstrated by the SAED (d) corresponding to the red circle in (c). e) HAADF image of aggregated blade-like MSU surrounded by NaCl crystals, along with their corresponding Na, N, and Cl EDS maps. Note that care must be taken to identify the different phases in SF samples, as they include large amounts of sodium chloride that might interfere in the identification using TEM. This is why precipitates were systematically analyzed using HAADF-EDS.

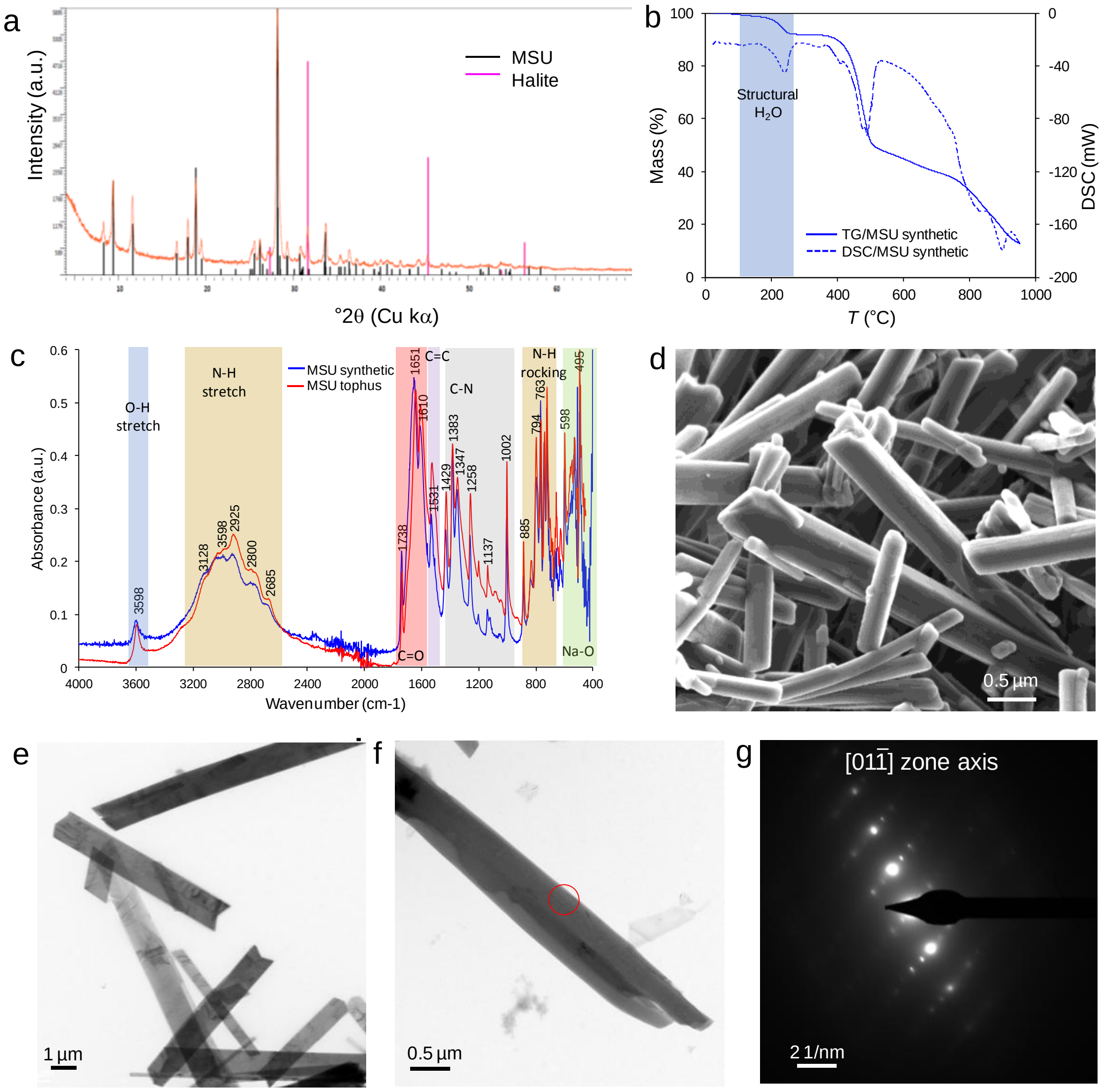

Fig. S5. **Characterization of abiotic MSU**. a) XRD synthetic MSU 4.5 days after titration synthesis. There is MSU (grey bars; JPDF 31-1890) plus halite (red bars JPDF 5-628). Corresponding TG-DSC (b), FTIR (c), FESEM (d) of MSU synthesized in titration experiments. e) TEM image of MSU synthesized by rapid reactants mixing. Note the abundant "fish-tail" twinning; f) TEM image and g) SAED pattern of gel-grown MSU.

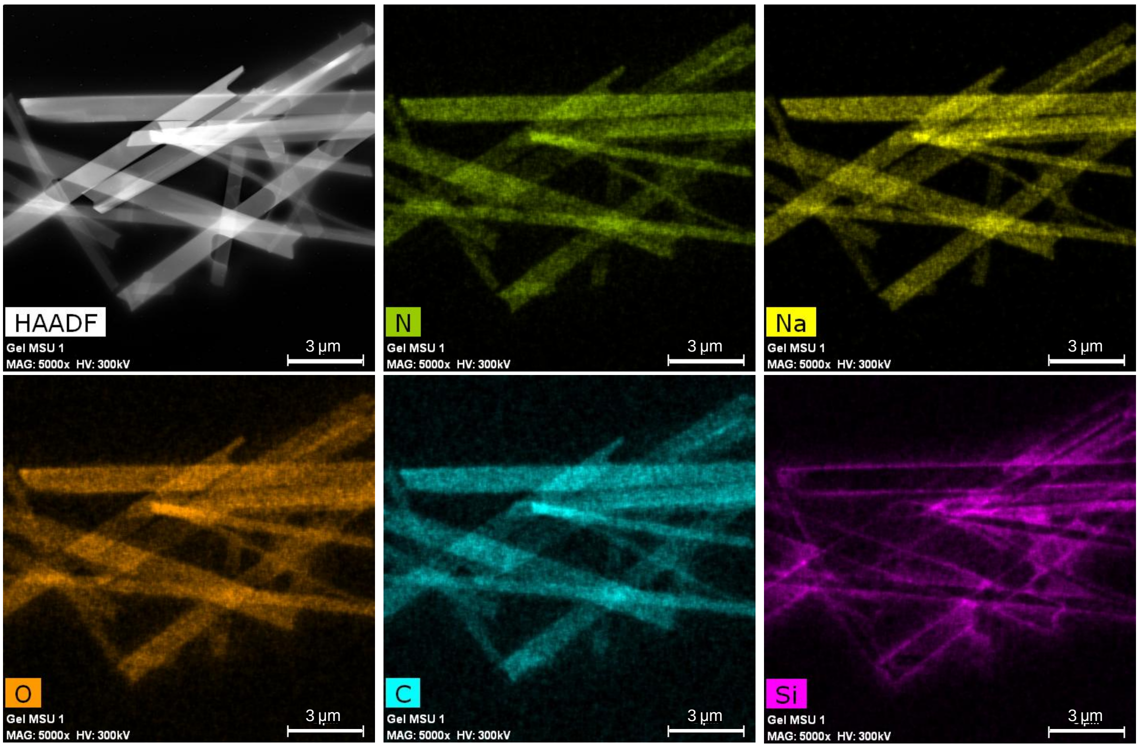

Fig. S6. **HAADF image and corresponding elemental EDS maps of gel-grown MSU crystals.** Note the presence of Si surrounding MSU crystals (the Si corresponds to silica gel).
