# Supplementary material for: Unraveling the pathological biomineralization of monosodium urate crystals in gout patients
Source: Commun Biol. 2024 Jul 7;7:828. doi: 10.1038/s42003-024-06534-6 (PMC11228021; doi:10.1038/s42003-024-06534-6)
Supplement: Supplementary file 3 — Description of Additional Supplementary Files [file 42003_2024_6534_MOESM3_ESM.pdf]

Description of Additional Supplementary Files

File: Supplementary data 1

Description: XRD data.

File: Supplementary Data 2

Description: FTIR and Raman spectroscopy data.

File: Supplementary Data 3

Description: TG-DSC data.

File: Supplementary Data 4

Description: Titration tests data.

File: Supplementary Data 5

Description: Synchrotron HRXRD data.
